# Distal Leg Posterior Tibial Nerve Schwannomas Combined With Tarsal Tunnel Syndrome: A Case Series and Literature Review

**DOI:** 10.7759/cureus.79356

**Published:** 2025-02-20

**Authors:** Gabriel Verly, Marcus André Acioly

**Affiliations:** 1 Neurosurgery, Federal University of Rio de Janeiro, Rio de Janeiro, BRA; 2 Neurosurgery, Federal Fluminense University, Niterói, BRA

**Keywords:** entrapment, nerve, posterior tibial nerve, schwannomas, tarsal tunnel syndrome

## Abstract

While the pathophysiology of space-occupying lesions inside the tarsal tunnel causing tarsal tunnel syndrome (TTS) is obvious, the occurrence of a posterior tibial nerve (PTN) mass outside the tarsal tunnel but mimicking or in combination with TTS symptomatology is less clear. Therefore, we report three rare cases of patients presenting with TTS symptoms combined with a distal leg PTN schwannoma, all of whom were treated with tumor resection and tarsal tunnel decompression. All patients had a long-lasting history of leg and ankle pain radiating to the medial aspect of the foot and toes. Pain was especially worsened at night, during walking, and with weight bearing. PTN schwannomas were located at the distal third of the leg. One patient was secondarily decompressed after previous tumor resection elsewhere. The surgical approach included tumor resection with fascicle-sparing enucleation and tarsal tunnel decompression with single or two incisions. Tumor resection was complete in all patients, and the diagnosis of schwannomas was confirmed by histological analysis. Transient-sensitive deterioration was documented in one patient, but there were no motor complications. The pain was resolved completely in two patients and partially in one patient at the last follow-up. PTN schwannomas mimicking or in combination with TTS make the diagnosis challenging since they share most of the clinical symptoms and signs. Such recognition is of utmost importance for better patient management. In these situations, tarsal tunnel decompression could be included in the surgical procedure to achieve long-lasting relief of pain.

## Introduction

Tarsal tunnel syndrome (TTS) refers to an entrapment neuropathy of the posterior tibial nerve (PTN) or its branches, as it passes through a narrow fibro-osseous tunnel caudal to the medial malleolus [1,2]. Even though TTS was recognized before [2,3], the term is attributed to Keck [4] and Lam [5] in two independent studies in 1962. TTS was initially introduced to describe PTN compression neuropathy of idiopathic causes, but more recently, the term encompasses other specific conditions [6]. Any disease that reduces the existing space inside the tunnel might produce PTN compression with the characteristic sequence of symptoms [1,2,7]. Thus, several space-occupying lesions of the soft tissues, extrinsic osseous deformity from the tarsal tunnel floor, and intrinsic PTN conditions, such as schwannomas, can cause and mimic TTS [8,9].

While the pathophysiology of space-occupying lesions inside the tarsal tunnel causing TTS is obvious, the occurrence of a PTN mass outside the tarsal tunnel, but mimicking or in combination with TTS symptomatology is less clear. Therefore, we report on three rare cases of patients with a mean age of 55 ± 10.2 years (range, 46-70 years), presenting TTS symptoms in combination with a distal leg PTN schwannoma. We aim to raise suspicion and recognition of such unusual combinations for better patient management. They were treated with tumor resection and tarsal tunnel decompression at our institutions over the last 12 years. This paper was previously presented as a meeting abstract at the XXXIV Brazilian Congress of Neurosurgery on September 29, 2023.

## Case presentation

Case 1 

**Figure 1:**
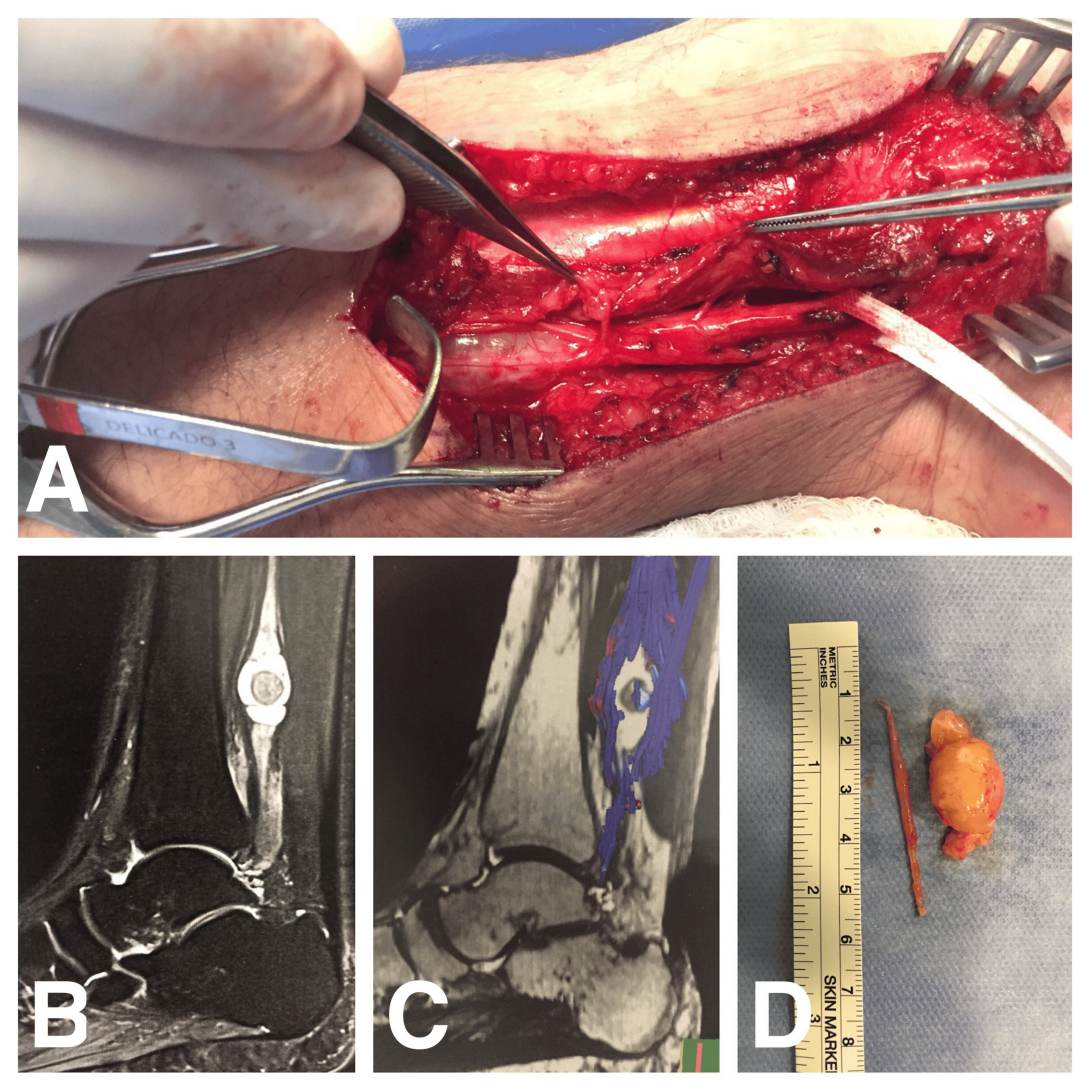
Case 1: Intraoperative views showing complete tarsal tunnel decompression and tumor resection (A, D); sagittal T2-weighted MRI (B) and MR neurography (C) images demonstrate a distal leg tibial nerve schwannoma displacing nerve fascicles laterally MRI, magnetic resonance imaging; MR, magnetic resonance

A 46-year-old male patient was referred for consultation due to intense and progressive pain in his left ankle and foot. His symptoms had been present for a year and were consistently worsening at night and after physical activity. Physical examination was difficult due to severe pain, but a tender mass with a positive Tinel sign was encountered at the distal third of the left leg. Toe flexion deficit and slight paresis were documented, but the rest of the physical examination was otherwise normal. Magnetic resonance imaging (MRI) revealed a 3.5-cm peripheral nerve sheath tumor (PNST) of the PTN proximal to the tarsal tunnel (Figure 1B). Neurophysiological examination revealed normal sensory and motor conduction velocities. The diagnosis of TTS could not be ruled out, so we discussed management options, including tumor resection or tumor resection combined with tarsal tunnel release. The patient opted for the combined surgery since he could no longer live with such debilitating pain, which was refractory to neuropathic pain medication. During surgery, the tarsal tunnel was completely released with a single incision and the tumor was completely resected via fascicle-sparing technique under electrophysiological guidance (Figures 1A, 1D). Histopathological examination confirmed the diagnosis of a schwannoma. The patient developed transient impairment of the heel sensory function with complete resolution of pain and motor function during the immediate postoperative period. Heel hypoesthesia was completely resolved at four months. At the last follow-up (87 months), the patient was asymptomatic, and complete tumor resection was documented by imaging. 

Case 2

**Figure 2:**
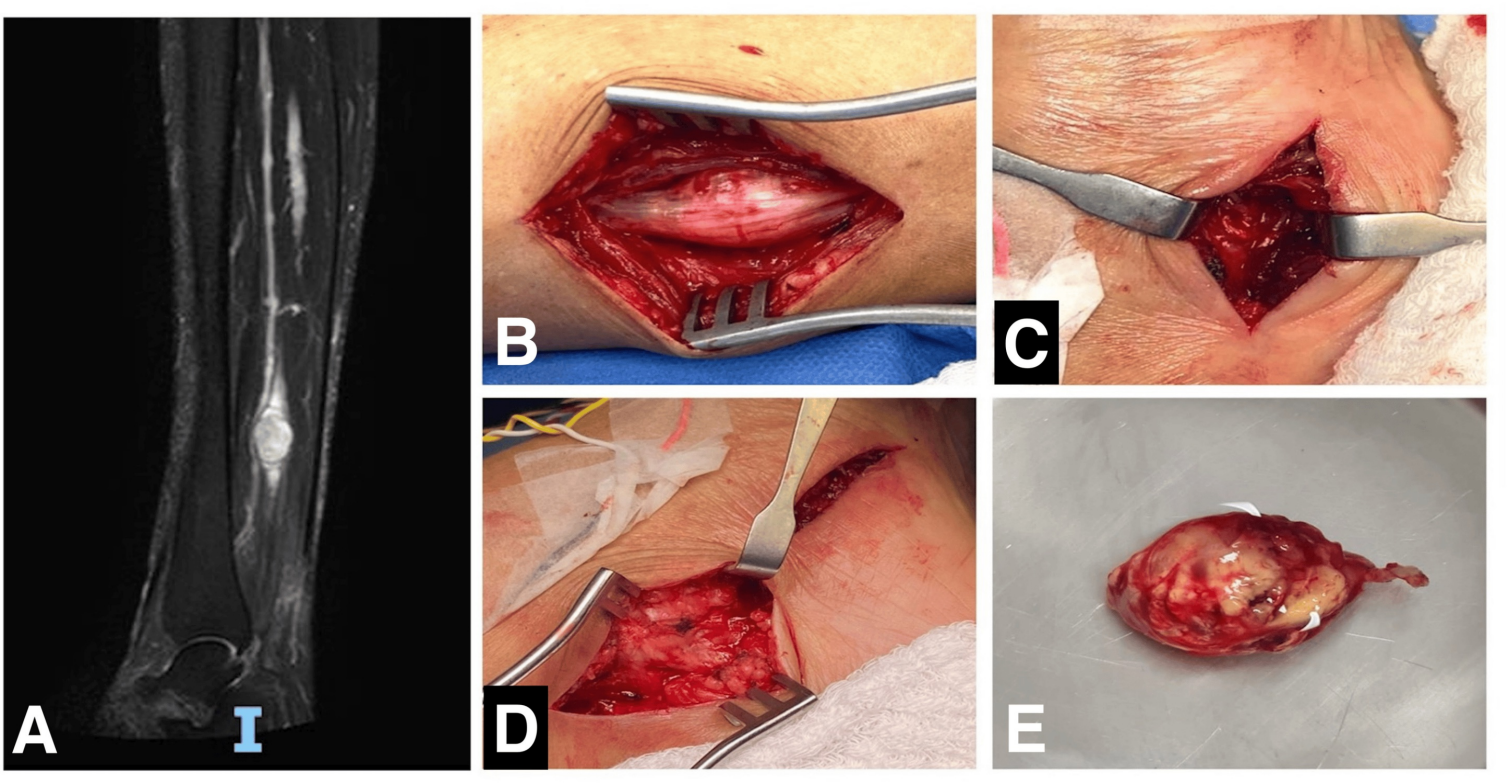
Case 2: Sagittal T2-weighted MRI showing a distal leg tibial nerve schwannoma (A); intraoperative views showing the tumor before gross total resection and complete tarsal tunnel decompression through two incisions (B-E). MRI, magnetic resonance imaging

A 52-year-old woman presented to our department with a 20-year history of left forefoot pain and a 4-year history of ankle and leg neuropathic pain. Her symptoms were initially attributed to an ipsilateral Morton’s neuroma. She developed intense and progressive pain that worsened at night and during activity. A palpable and tender mass was noted at the distal third medial aspect of the left leg. The mass evoked a positive Tinel sign, and she also had a positive tarsal tunnel compression test. Neurological examination revealed the reduced motor function of the abduction and flexion of the little toe (Medical Research Council [MRC] grade 4), while sensory testing showed hypoesthesia on the lateral aspect of the foot sole (no temperature sensation). MRI showed a 2-cm diameter PNST at the distal leg. PTN imaging within the tarsal tunnel was otherwise normal. Neurophysiological examination demonstrated no sensory action potential of the left lateral plantar nerve, highly suggestive of TTS. The electromyography (EMG) pattern revealed no abnormalities. After careful discussion with the patient, we decided to undergo tumor resection and tarsal tunnel decompression under electrophysiological guidance (Figures 2B-2E). The patient did very well postoperatively, experiencing complete resolution of pain, full motor recovery, and partial sensory recovery, with slight hypoesthesia on the lateral aspect of the foot and complete recovery of temperature sensation. Histopathological examination confirmed the diagnosis of a schwannoma. Complete tumor excision was confirmed with postoperative MRI. She experienced residual ankle edema, which was completely resolved at the last follow-up (27 months). 

Case 3 

**Figure 3:**
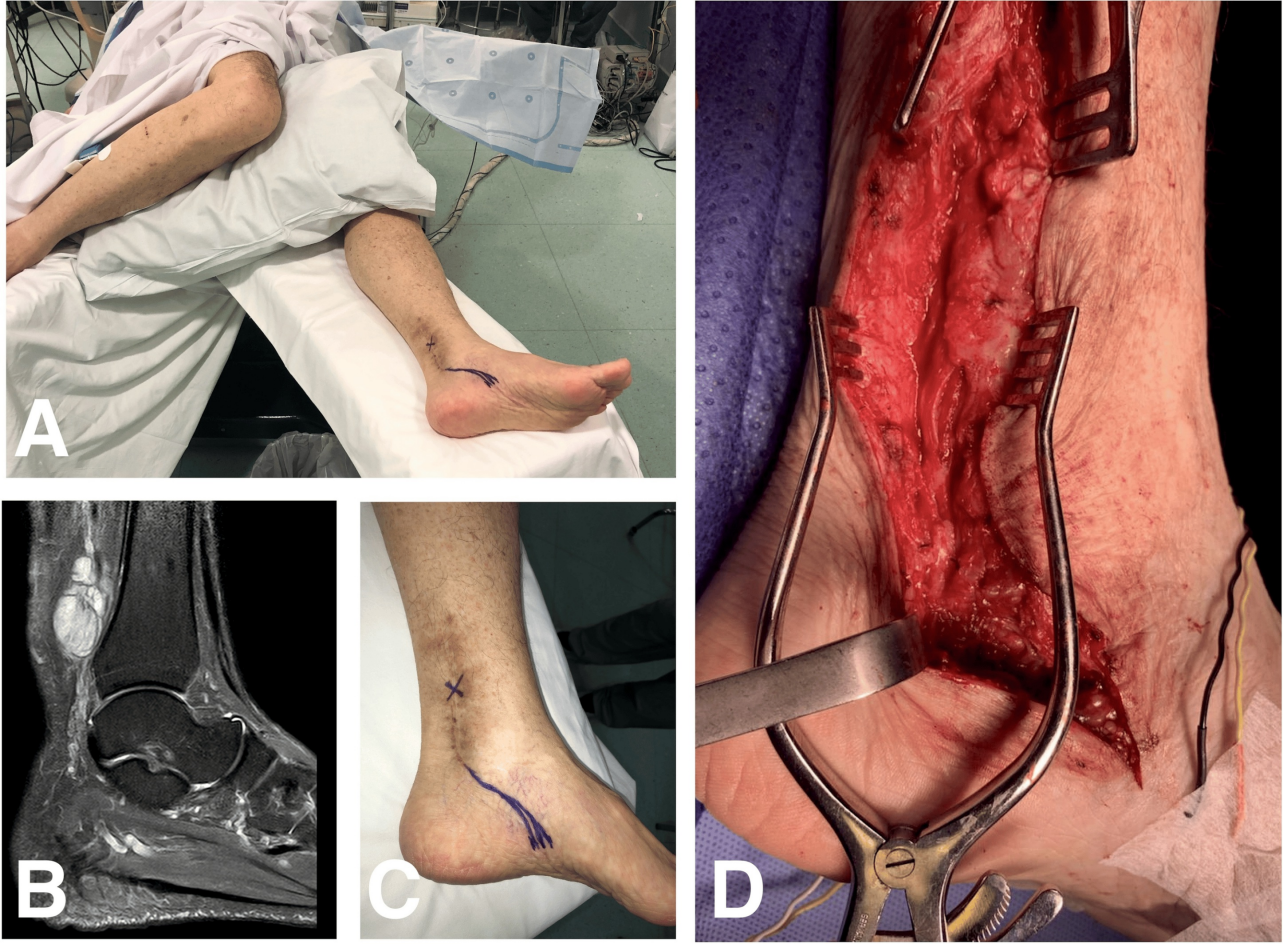
Case 3: Intraoperative photographs showing surgical positioning and planned skin incision, as well as the final aspect of tarsal tunnel decompression (A, C, and D); sagittal T2-weighted MRI of the left leg three years before the current admission demonstrating a distal leg tibial nerve schwannoma outside the tarsal tunnel (B).

A 70-year-old gentleman presented to our department with persistent neuropathic pain in the left ankle and foot following a distal leg schwannoma resection performed elsewhere three years before his current admission. Foot paresthesia had been present for 20 years, while pain started four years before the aforementioned surgery. He underwent a second revision surgery elsewhere, including internal neurolysis to address residual pain, but had no improvement. He experienced daily pain that worsened at night, especially at rest. During his current admission, a neurological examination revealed slight weakness in toe flexion (MRC grade 4) and heel numbness. Positive Tinel sign was elicited over the tarsal tunnel. MRI revealed diffuse posterior tibial nerve enlargement and high signal on T2-weighted images inside the tarsal tunnel. Electrophysiological studies were not performed preoperatively. Diagnosis of TTS was established, and we offered tarsal tunnel decompression. During surgery, the tarsal tunnel was completely released under intraoperative neurophysiological monitoring with a single incision (Figure 3). Intraoperatively, neurophysiological monitoring before TTS decompression revealed a complete motor conduction block. Thereafter, motor potentials recovered with lower amplitude. Overall, the outcomes included partial resolution of pain, with a 56% improvement in the visual analog scale (VAS) score (decreasing from 70 to 30 postoperatively), complete motor recovery, and partial sensory recovery, with slight heel hypoesthesia at the last follow-up. There were no postoperative complications. At the last follow-up (40 months), the patient still experienced some residual ankle and foot pain. Spinal cord stimulation was offered, but the patient declined surgical indication.

## Discussion

Schwannomas are benign PNSTs that arise from the Schwann cells and fibroblasts as a well-encapsulated slow-growing mass within the nerve bundle [8-10]. While a recent large study reported a relatively even distribution of schwannomas across the brachial plexus and upper and lower extremities [11], other authors consider lower limb involvement, particularly of the PTN, to be rare [8-10]. About 10% of all PNSTs occur in the ankle and foot, with schwannomas representing 57% of these cases [12]. 

Neuropathic pain is, in practical terms, a *sine qua non* condition of PNSTs, as approximately 98% of patients develop moderate pain (VAS between 4 and 6) [11]. Characteristically, pain is generally evoked after tapping the region of the lump. On the other hand, patients with TTS generally complain of intermittent burning pain, tingling, or numbness in the foot, which is exacerbated by activities such as prolonged standing or walking [13,14]. Pain in the context of TTS typically worsens at night [13,14]. A possible hypothesis is that the nocturnal worsening of TTS results from fluid redistribution, reduced circulation, inflammatory processes, mechanical compression, and heightened nerve sensitivity, collectively exacerbating neuropathic pain. Objective foot sensory loss and motor weakness of the toe abductors and flexors, along with a positive Tinel sign over the tarsal tunnel, are commonly reported in the literature. However, precise clinical and electrophysiological criteria remain widely lacking [14].

The American Association of Neuromuscular and Electrodiagnostic Medicine (AANEM) provided evidence-based recommendations for electrodiagnostic techniques, suggesting that prolonged distal latencies in PTN motor conduction studies and slowed conduction velocities across the tarsal tunnel in sensory conduction studies are diagnostic for suspected TTS [14]. The utility of needle EMG remains uncertain. Notably, about one-third of patients with TTS have normal electrophysiological studies [1]. Accordingly, the role of electrophysiological studies in PNSTs is limited, as they are generally normal [6,11].

Thus, PTN schwannomas and TTS might have overlapping symptoms, even for PNSTs outside the tarsal tunnel. This assumption is not new, as Padua et al. [15] previously noted and described five patients with median nerve schwannomas at the arm, elbow, forearm, wrist, and palm that mimicked carpal tunnel syndrome. Notably, two patients had previously undergone carpal tunnel release without any improvement. In our literature review, we identified an additional five patients with PTN schwannomas in the distal leg presenting with symptoms resembling TTS. Patients’ details are provided in Table 1 [1,6,10,16,17]. Preoperative electrodiagnostic studies were done on only two patients. Solely one patient had increased latency in nerve conduction studies over the tarsal tunnel [16]. Schwannoma resection was performed in three patients, while the other two underwent tumor resection in combination with tarsal tunnel release. Surgical outcomes were generally successful, as all patients were asymptomatic at the last follow-up.

**Table 1:** Reported cases of PTN combined with TTS in the literature. ^*^Two- or three-dimensional information was considered. No., number; mo., month; F, female; M, male; NR, not reported; MRI, magnetic resonance imaging; USG, ultrasound; EMG, electromyography; NCS, nerve conduction studies; TTS, tarsal tunnel syndrome; PTN, posterior tibial nerve

Study	No. of cases	Sex	Age (years)	Clinical presentation	Pain duration	Electrophysiological studies	Imaging	Tumor size*	Management	Outcome
Belding (1993) [6]	1	F	39	Progressive retromalleolar pain accompanied by burning and tingling in the plantar aspect of the sole, with a positive Tinel sign over the tibial tunnel (TT).	1 year	Sensory and motor latency within normal range; no fibrillation	MRI	1.2 cm x 1.5 cm	Surgical resection; no mention of TT release	Symptom-free at 1 year postoperative
Jha et al. (2019) [10]	1	M	71	Shocking left foot and ankle pain	6 years	NR	MRI	3.0 cm x 2.0 cm x 1.5 cm	Surgical resection and TT release	Symptom-free at two years postoperative
Rajasekaran and Shanmuganathan (2018) [16]	1	M	58	Pain over the medial aspect of the right foot associated with occasional numbness over the plantar aspect of the foot	1.5 years	NCS with increased latency of the posterior tibial nerve with a reduction in amplitude of motor unit.	MRI	1.3 cm x 1.0 cm	Surgical resection	Symptom-free at 6 mo postoperative
Schweitzer et al. (2013) [17]	1	M	63	Progressive pain associated with activity	3 mo	NR	MRI	2.8 cm	Surgical resection; no mention of TT release	Symptom-free at 1 year postoperative
Tladi et al. (2017) [1]	1	M	46	Medial ankle and foot pain radiating to the lateral aspect of the sole	15 years	NR	USG	2.8 cm x 2.0 cm x 1.8 cm	Surgical resection; TT release	Symptom-free at 8 weeks postoperative
This study	3	M	46	1 year of ankle and sole pain	1 year	Normal sensory and motor conduction velocities	MRI	NR	Surgical resection; TT release	Complete resolution of pain, and complete motor recovery
		F	52	20 years of forefoot pain, 4 years of leg and ankle neuropathic pain	20 years and 4 years	Sensory and motor latency within normal range	MRI	2 cm x 1.8 cm x 1.7 cm	Surgical resection; TT release	Complete resolution of pain, complete motor recovery, and partial sensory recovery
		M	70	20 years of forefoot paresthesias, and 4 years of leg and ankle neuropathic pain	20 years and 4 years	Not performed	MRI	5 cm x 3 cm	TT release	Partial resolution of pain, complete motor recovery, and partial sensory recovery

On the other hand, our experience, along with the observations of Padua et al. [15], suggests that some patients may experience residual pain after isolated tumor resection or anatomical tunnel release due to the combination of a PNST outside anatomical fibro-osseous tunnels and symptoms of entrapment neuropathy. In such cases, it is of utmost importance the identification to improve patient management. Pathogenesis is widely unknown, but we suggest the same mechanism as the double-crush syndrome, in which a proximal lesion turns the distal nerve trunk more vulnerable to compression. Such a syndrome most likely represents disturbances in axonal flow kinetics and the disruption of the neurofilament architecture [18].

## Conclusions

PTN schwannomas and TTS share most of the clinical symptoms and signs, making diagnosis challenging. This is a rare clinical condition that might resemble TTS symptoms or may occur in combination. In these situations, our results support the inclusion of tarsal tunnel decompression in addition to tumor resection due to favorable outcomes and low complication rates.
